# Epidemiology of interpersonal violence at a regional hospital emergency unit in the Eastern Cape, South Africa

**DOI:** 10.4102/safp.v64i1.5511

**Published:** 2022-05-24

**Authors:** Amitabh Mitra, Uchenna B. Okafor, Ramprakash Kaswa, Oladele V. Adeniyi

**Affiliations:** 1 Department of Family Medicine, Faculty of Health Sciences, Walter Sisulu University, Mthatha, South Africa; 2 Department of Nursing Sciences, Faculty of Health Sciences, University of Fort Hare, East London, South Africa

**Keywords:** assault, Eastern Cape, emergency department, interpersonal violence, South Africa

## Abstract

**Background:**

This study describes the profile, mechanism and pattern of injuries, and highlights important gaps in clinicians’ consultations with patients who experienced interpersonal violence (IPV) in the predominantly black South African township of Mdantsane, Eastern Cape.

**Methods:**

This retrospective cross-sectional study was conducted at the Cecilia Makiwane Regional Hospital, Mdantsane. Medical records of patients who received emergency care for trauma between 01 December 2017 and 31 March 2018 were reviewed. The records of patients identified with IPV were selected for further analysis. Data were disaggregated by demographics, mechanism of injuries and circumstances of the incidents using simple descriptive statistics.

**Results:**

A total of 1064 patients reported IPV as the mechanism of injury for emergency department (ED) visits, accounting for 42.4% of all trauma-related injuries. The majority of patients with IPV were men (72.0%), unemployed (78.0%) and single (89.0%). Blunt force injury was the most common pattern of injury (53.3%); about half (50.5%) of the incidents took place in the patients’ homes. The majority of the patients (68%) knew their assailants, and a quarter of them were an intimate partner of the assailant (27.6%). The flow of patient with IPV to the ED was skewed towards the weekend (weekend effect). Also, there was an upward trend in the flow of patients with IPV to the ED from 19:00 onwards, reaching a peak at 20:00.

**Conclusion:**

Interpersonal violence is the most prevalent mechanism of injury reported in this region. It is crucial to engage stakeholders in the design of interventions in order to reduce IPV-related injuries in the region.

## Introduction

Interpersonal violence (IPV) is a serious public health issue that may affect anyone and known to kill thousands of people annually in most parts of the world.^1,2^ Interpersonal violence is a prevalent mechanism of trauma-related injuries, frequently leading to disability and death.^3,4^ The effects of this type of violence extend beyond the individual, thereby also affecting the costs of healthcare globally.^5^ It can also lead to social unrest, economic decline and violence against women.^3^

According to the World Health Organization’s (WHOs) Global Health Estimates, more than 1.3 million people died every year because of violence-related injuries, and out of these, about 35.3% died as a result of IPV.^4,6^ The United Nations continues to lead awareness campaign towards eliminating IPV globally.^1^ Interpersonal violence is defined by WHO as ‘the deliberate use of force or power, threatened or actual, against oneself or another person or against a group or a community’.^7^ This encompasses intimate partner violence and community violence. The consensus is that IPV is poorly reported globally, especially in low- and middle-income countries (LMICs), where 90% of death are attributed to trauma. A lack of reliable data, therefore, impacts prevention efforts for IPV-related injury.^4,7^

Physical violence is known to have negative effects on various health conditions: mental health, infectious diseases, substance abuse and non-communicable diseases.^4,8^ Some of the physical injuries sustained during IPV include lacerations, bruises and fracturesthat can lead to disability, such as brain damage or paralysis.^3,9^ Interpersonal violence increases the risk of mental health issues, such as depression and substance abuse during pregnancy.^10^ Children who experience childhood violence are more prone to having HIV than the general population. Being abused as a child can also increase the risk of becoming an adult victim or perpetrator of sexual violence.^11^ Survivors of IPV are at increased risk of psychological and behavioural problems, such as alcohol abuse, depression, post-traumatic stress disorders and suicidal behaviours.^2,3,11,12^

Violence-related injuries often go unreported. Several individuals and societal reasons have been advanced for the underreporting of IPV in developing countries.^4^ Often, injuries resulting from IPV may not be severe enough to warrant hospital visits, with such cases being underreported.^8^ However, when individuals suffer significant injuries, fear of revenge following the report of the perpetrators to authorities may preclude disclosure. In addition, societal norms and inefficient medical record systems contribute to a lack of reliable data on IPV.^8,12,13^

Evidence shows that injuries from IPV are a major contributor to the burden of trauma in South Africa.^14^ In addition, South Africa is among the countries with a particularly high rate of IPV, leading to serious morbidity and mortality rates.^11,15^ In order to successfully reduce the morbidity and mortality associated with traumatic injuries, accurate epidemiological data are needed. Specifically, understanding the context in which IPV thrives will help to guide policies on the prevention and the promotion of peaceful co-existence at the community level. This study, therefore, describes the profile of survivors, the timing of reporting after trauma and the circumstances preceding the injuries in patients who accessed a peri-urban regional hospital emergency department (ED) in the Eastern Cape province. These findings will be crucial for the crafting of effective regional intervention programmes and also serve as reference data for future evaluations of the effectiveness of the instituted intervention programmes.

## Method

### Study design

We conducted a retrospective cross-sectional study at ED of Cecilia Makiwane Hospital in Mdantsane, Eastern Cape, South Africa between 01 December 2017 and 31 March 2018. Cecilia Makiwane Hospital is a regional academic hospital, affiliated with Walter Sisulu University for the training of undergraduate and postgraduate medical students and serves a population of 755 500 in Buffalo City Municipality (BMC) in the Eastern Cape province. This ED renders acute care services for critically ill or injured patients either as the first point of care or as referrals from the entire municipality. It renders a 24-hour service with an efficient triage system using the South African Triage Scale^16^ to prioritise patients based on the severity of the presentation of the clinical condition.

### Participants and procedure

We retrieved a total of 2506 medical records of patients who received acute care for injuries in the emergency room during the study period from the hospital registry. The medical records were reviewed critically to identify patients who reported any form of violence without any restrictions by age or setting of injury. Overall, medical records of 1064 patients who met our inclusion criteria were included in this study. The research nurse extracted relevant data from the medical records of each patient using a data collection form designed specifically for this study. The data collection form consists of two parts: demographic data (age, gender, marital status and employment status) and injury-related data (time of injury, geographical location of the event, mechanism of injury, time of reporting to ED and circumstances preceding the injury).

### Variable definitions

In this study, IPV was defined as any form of violence leading to injury and consequent ED visits for acute care. It included all forms of physical injuries, in which one person or a group of persons caused injury to another. In addition, the use of weapons or objects as the mechanism of injury was documented. Where the objects or weapons used in inflicting injuries were not documented by the attending doctor. mssing data were reported as part of the key areas for quality improvement in the department.

Time of event was obtained from the clinical notes of the attending doctor at the first consultation in the ED. The time of seeking acute care following IPV (day of the week and time) was extracted from the medical records. Where neither of these data were available, missing data were recorded and reported accordingly.

### Statistical analysis

Data were analysed using the Statistical Package for Social Sciences (SPSS, version 27.0) for Windows (IBM Corp., Armonk, New York, United States). Baseline characteristics of the participants were presented as categorical variables with frequencies and percentages. The pattern of events surrounding the cases of IPV, days of IPV event and the estimated time of reporting to the ED following IPV event were analysed using simple descriptive statistics.

### Ethical considerations

Ethical approval was obtained from the Walter Sisulu University Human Research and Ethics Committee (Reference number: 064/2017). In addition, permission was granted by the Eastern Cape Department of Health and the clinical governance of the hospital (064/2017, 30 October 2017).

As this is a retrospective review of medical records, no additional permission was obtained from the patients. However, the research team ensured anonymity and confidentiality of medical information during and after the study. Implementation of the protocol followed the Helsinki Declaration and principles of good clinical practice governing human research.

## Results

Overall, a total of 1064 participants reported IPV, which accounted for 42.5% of all trauma cases managed during the study period. The majority were men (71.9%), aged between 25 and 44 years (60.9%), single (89.1%) and unemployed (77.8%) (Table 1).

**TABLE 1:** Sociodemographic characteristics of the participants.

Variables	Frequency	Percentage
**Gender (*n* = 1064)**		
Male	765	71.9
Female	299	28.1
**Age (years) (*n* = 1060)**		
0–14	41	3.9
15–24	218	20.5
25–34	420	39.5
35–44	228	21.4
45–54	97	9.1
55 and above	56	5.3
**Employment status (*n* = 410)**		
Unemployed	319	77.8
Employed	56	13.7
Student	35	8.5
**Marital status (*n* = 604)**		
Single	538	89.1
Married	53	8.8
Windowed	10	1.6
Divorced	3	0.5

### Geographical location, circumstances and mechanism of interpersonal violence

The mechanism of injury was documented by the attending doctors in 83.6% of the cases (*n* = 889). In most of the cases, the object used for inflicting injuries include blunt (53.3%) followed by knife resulting in stab injury (45.3%), sjambok (0.8%) and gunshot (0.6%). Assault (one person attacking another) was the predominant circumstance (90.7%) leading to injury, followed by physical fight (two or more people attacking each other) (6.6%) and robbery (2.7%).

The geographical location where IPV took place was the least documented by the attending clinician (*n* = 212). Of the locations recorded, IPV incidents were more prevalent at home (50.5%), followed by the streets (27.4%), while the rest took place in different parts of the community (Table 2).

**TABLE 2:** Mechanisms of injury, circumstances and geographical location of interpersonal violence.

Mechanisms of injuries in IPV events (*n* = 889)	Frequency	Percentage
Blunt injury	474	53.3
Stab injury	403	45.3
Sjambok injury	7	0.8
Gun shot	5	0.6
**Circumstances of IPV (*n* = 594)**		
Assault	539	90.7
Fight	39	6.6
Robbery	16	2.7
**Geographical location (*n* = 212)**		
Home	107	50.5
Street	58	27.4
Tavern	20	9.4
Work	10	4.7
School	7	3.2
Community	6	2.8
Gym	1	0.5
Club	1	0.5
Prison	1	0.5
Shopping mall	1	0.5

IPV, interpersonal violence.

Note: Sjambok is a heavy leather whip, made from animal’s hide.

### Relationship between interpersonal violence survivors and assailants

Two-thirds of survivors (67.9%) knew their assailants. Among these known perpetrators, the spouse (partner) was responsible for a quarter (27.6%) of cases, followed by family members (19.4%) and friends (9.3%). Employers and co-workers, community mobs and other community members accounted for the remaining cases (Table 3).

**TABLE 3:** The relationship between victims and assailants.

Familiar to the patients (*n* = 377)	Frequency	Percentage
Unknown persons	121	32.1
Partner	104	27.6
Family member	73	19.4
Friend	35	9.3
Employers and co-workers	14	3.7
Community mob	14	3.7
Neighbours	7	1.8
Policemen	6	1.6
Nanny	2	0.5
Teacher	1	0.3

### Timing of reporting of interpersonal violence events to the emergency department

**FIGURE 1:**
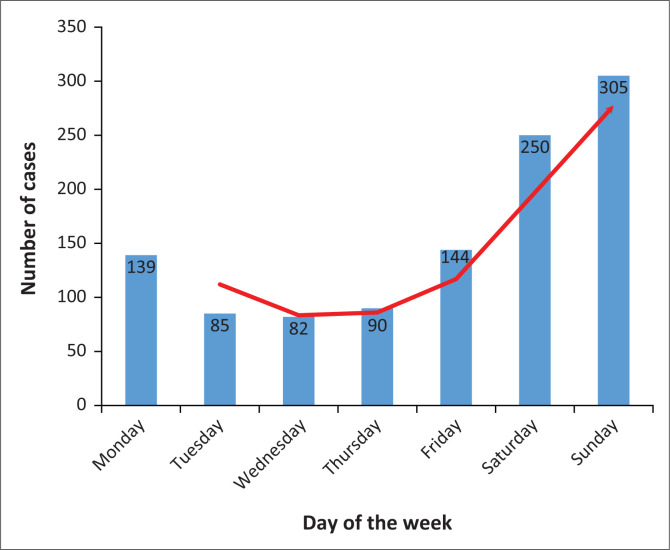
Trends of daily visits to emergency department following interpersonal violence.

There was a significant weekend effect in the utilisation of ED by victims of IPV, with a gradual increase in the incidence of IPV from Thursdays to Sundays, with the peak number reached on Sundays. This was followed by a steep decline in the incidence of IPV from Mondays to Wednesdays (Figure 1).

**FIGURE 2:**
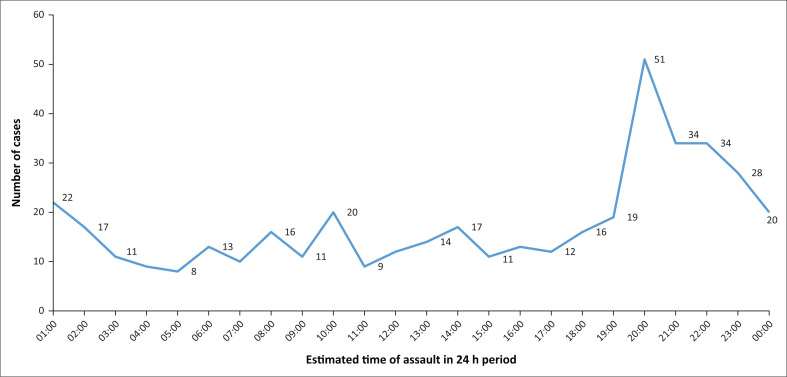
Estimated time of emergency department visits following interpersonal violence.

The highest influx of IPV victims to ED for acute care occurred around 20:00, followed by a sharp decline until midnight. After midnight, the flow of IPV patients remained steady during the whole day until 19:00, followed by a sharp increase until 20:00 (Figure 2).

## Discussion

Interpersonal violence is a leading contributor to the overall trauma burden and mortality globally.^2,4^ Trauma-related injuries attributed to IPV in LMICs are 10 times higher than those in high-income countries.^3^ However, a lack of regional data preclude appropriate planning for strategies to mitigate the burden of IPV in South Africa. This study reports the first epidemiological data on IPV-related injuries from the Eastern Cape, South Africa. We found that a significant proportion of the total trauma burden at this emergency unit (42.5%) was attributed to IPV. This is in agreement with previous reports.^11,14^ Data collected by South Africa’s National Injury Surveillance System and Death Certificates show that 48.0% of the country’s deaths are caused by violence.^17^

This study reports a male predominance (71.9%) in comparison with female. This finding is in agreement with previous reports showing male predominance among survivors of IPV.^6,9^ While the rate among women may be underreported, given that IPV is self-reported. Nonetheless, this finding supports previous studies that have reported a higher rate among men (3.5:1).^9,13^ This study also found that almost two-thirds (60%) of violent episodes occurred among young adults, with the age group being generally 15–34 years. Young people are more prone to experience violence than their older counterparts. A study conducted in 2013 revealed that about 44% of victims of physical assaults were under the age of 25 years.^11^ A plausible explanation for this result could be that young adults often like to assert their independence by acting out violently.

The majority of the survivors suffered from blunt force injuries, which are usually less severe than penetrative injuries. This finding is in agreement with results from a Ugandan study, which showed that blunt force injuries were the predominant trauma experienced among patients.^5^ In contrast to developed countries, where the majority of trauma admissions are a result of self-inflicted injuries, in developing nations, blunt force trauma is the norm.^4^ In addition, the study reports assaults, robberies and physical fights as the circumstances leading to IPV in this cohort. These types of violent acts frequently end with the death of economically productive young people.^3,5^ Hence, prompt reporting of these violent acts by the community members to the police might be effective in reducing such incidents in the region.

In most cases, the incidents took place in the victims’s home and the survivors knew their assailants. Other studies have found that the attacker is frequently the spouse or partner of the survivor.^3,4,8^ This study reported similar findings; in cases in which the assailants were known (67.9%), about a quarter of them (27.6%) were the intimate partners of the victims. A random survey conducted in South Africa in 2002 revealed that almost 30% of women in the country have experienced physical assault from their partners.^11^ Most of the young men who visit the study setting experienced trauma during weekends because this type of violence occurs mostly at night and on weekends. The increased number of IPV incidents during weekends has been attributed to the ‘weekend effect’^9^, which involves an increase in social interactions.^5,11^ At night, most victims go to the emergency room for help, with numbers dropping off in the early hours of the morning. Aside from unemployment and high-income inequality, South Africa’s high rate of alcohol consumption is known to contribute to generally high levels of violence.^2,17,18^

## Limitations of the study

This study highlights important information on IPV in the region, as well as gaps in clinicians’ documentation in the medical records of patients. The limitations of the study cannot be ignored. The retrospective cross-sectional nature of the study did not allow for direct interviews of the participants and several missing data on important measures. In addition, this study used data of patients who sought emergency care in a hospital after IPV; the rate obtained in this study may well be the tip of the iceberg of those who suffer from IPV in the region.

## Conclusion

Interpersonal violence-related injuries among young adults aged 15–34 years, especially among economically productive adult males, are a leading cause of ED visits in the region. The mechanism of injury among the majority of patients is blunt force, largely resulting from physical assault, which often takes place at home (domestic violence) or in the community. The majority of victims knew their assailants, and most injuries were reported during weekends. It is crucial to engage stakeholders in the design of interventions to reduce IPV-related injuries in the region. Documentation of the victims and their injuries was found to be incomplete in many cases. Comprehensive documentation of clinical notes by attending clinicians is paramount to improve the quality of service delivered to the population. As such, the management should consider implementing a quality improvement project to strengthen documentation in the medical records of patients by the attending clinicians.
